# External cervical resorption detected via cone‐beam computed tomography in a patient with myelin oligodendrocyte glycoprotein antibody–associated disease: A case report

**DOI:** 10.1002/ccr3.4415

**Published:** 2021-07-06

**Authors:** Syuichi Munenaga, Momoko Usuda, Kazuhisa Ouhara, Yuta Maetani, Mikihito Kajiya, Shinji Matsuda, Hisako Furusho, Mutsumi Miyauchi, Masahiro Nakamori, Hirofumi Maruyama, Hiromi Nishi, Hiroyuki Kawaguchi

**Affiliations:** ^1^ Department of General Dentistry Hiroshima University Hospital Hiroshima Japan; ^2^ Department of Periodontal Medicine Graduate School of Biomedical and Health Sciences Hiroshima University Hiroshima Japan; ^3^ Department of Clinical Neuroscience and Therapeutics Graduate School of Biomedical and Health Sciences Hiroshima University Hiroshima Japan; ^4^ Department of Oral and Maxillofacial Pathobiology Graduate School of Biomedical and Health Sciences Hiroshima University Hiroshima Japan

**Keywords:** cone‐beam computed tomography, demyelination, external cervical resorption, myelin oligodendrocyte glycoprotein

## Abstract

External cervical resorption may occur in patients with MOG antibody–associated disease, which is clearly detected on cone‐beam computed tomography. Therefore, dental screening is essential for these patients before initiating bisphosphonate therapy. Larger sample sizes are crucial to determine any possible association between external cervical resorption and MOG antibody–associated disease.

## Graphical abstract

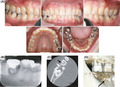

## 1 INTRODUCTION

External cervical resorption may occur in patients with MOG antibody–associated disease. Herein, we present the first case in the literature. Cone‐beam computed tomography is useful for the diagnosis of external cervical resorption in patients with MOG antibody–associated disease that would otherwise be undetected via radiography.

Myelin oligodendrocyte glycoprotein (MOG) is the main protein component of the myelin sheath in the central nervous system (CNS).^1^ MOG antibody–associated disease is a rare, autoimmune disorder that targets MOG, predominantly affecting the myelin in optic neuritis and myelitis, which can lead to vision loss and paralysis. Immunosuppressive therapies, such as steroids treatment, are often required for the treatment of MOG antibody–associated disease.^2^, ^3^ However, patients who receive long‐term steroid treatment require considerable monitoring owing to the risk of osteoporosis, a common side effect of steroids.

Bisphosphonate (BP) agents have been widely employed as a pharmaceutical therapy to prevent steroid‐induced osteoporosis in patients with MOG antibody–associated disease.^4^, ^5^ They are considered an integral component that supports the clinical efficacy and safety of long‐term steroid therapy. Unfortunately, this promising antiresorptive medicine also induces serious adverse effects, such as medication‐related osteonecrosis of the jaw (MRONJ). MRONJ is an emerging oral complication characterized by refractory bone exposure in individuals undergoing antiresorptive therapy.^6^ Since bone manipulation, such as tooth extraction, is an important trigger for MRONJ, patients should undergo a comprehensive dental examination before starting BP therapy.^7^, ^8^

External cervical resorption (ECR) is the loss of dental hard tissue as a result of odontoclastic action.^9^ There are various causes of ECR, including extraction of a neighboring tooth, malocclusion, playing wind instruments, periodontitis, autotransplantation, transmission of feline viruses to humans, herpes zoster, systemic and genetic factors, the use of bisphosphonates, impacted teeth, cysts, tumors, and pressure of erupting canines on the lateral incisors. When ECR is extensive, the extraction of the affected tooth may be the only treatment.^10^ Therefore, before considering the use of a BP agent, dental examination is needed to locate ECR lesions. ECR has been reported in patients with autoimmune diseases, such as systemic scleroderma.^11^, ^12^, ^13^ However, as far as we know, ECR of MOG antibody–associated disease has not yet been reported in current literature. The objective of this article was to describe a case of MOG antibody–associated disease accompanied by ECR, in which cone‐beam computed tomography (CBCT) was useful for diagnosis.

## 2 CASE HISTORY/EXAMINATION

The patient was a 33‐year‐old Japanese man without notable personal or familial medical history, and medicine intake. The patient presented to the hospital suffering from mild but subacute progressive numbness of the neck and trunk. Physical examination showed no dysfunction of cranial nerves, muscle weakness, or cerebral ataxia, but dysesthesia and sensory disturbance in the region under the third cervical cord level were observed. Nerve conduction test and whole‐body computed tomography revealed no abnormal findings. However, magnetic resonance imaging of the head revealed swelling of the medulla oblongata and a T2 high‐intensity lesion with a contrast effect on the right dorsal side of the medulla oblongata. Total protein and myelin basic protein were elevated in the cerebrospinal fluid, and laboratory tests revealed no antibodies of aquaporin 4 or collagen disease but were positive for MOG antibodies. Subsequently, the patient was diagnosed with MOG antibody–associated disease, and treatment with long‐term oral steroids and a BP agent was planned. Before the initiation of BP treatment, a close examination of the oral cavity was performed.

**FIGURE 1:**
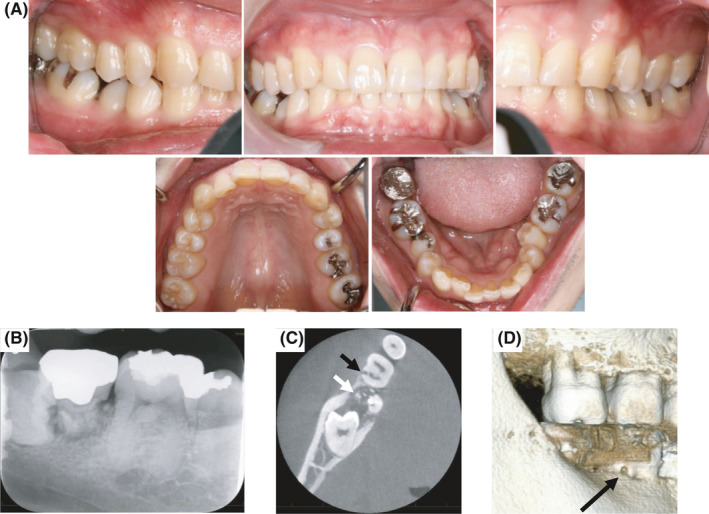
Photograph and radiogram of the oral cavity on the first examination. A, Photograph of the oral cavity, showing that the occlusion of the full cast crown of the right mandibular second molar had already been adjusted and the occlusal surface had become flat. B, Dental radiograph, indicating external root resorption in the right mandibular second molar. C, Axial view of cone‐beam computed tomography (CBCT), showing external root resorption in the right mandibular second molar (white arrowhead) and external cervical resorption on the buccal surface of the distal root of the right mandibular first molar (black arrowhead). D, Three‐dimensional reconstruction of CBCT, showing the resorption cavity on the buccal surface of the distal root of the right mandibular first molar (black arrowhead)

The patient underwent a dental examination after developing right mandibular gingival pain approximately 6 months before the first visit. He had no history of traumatic injury, internal bleaching, surgery, bruxism, or orthodontic treatment. In the first examination of the oral cavity, restorative teeth were found attached to the molars. There was no obvious caries or periodontal disease (Figure 1A). Dental radiography revealed a horizontally impacted right mandibular third molar and right mandibular second molar with marked root resorption, and extraction of these two teeth was planned (Figure 1B). Upon close examination using CBCT, ECR was incidentally noted on the buccal surface of the distal root of the right mandibular first molar (Figure 1C, D). CBCT examination (3D Accuitomo F17 MCT‐HN; J. Morita Corp) was performed by using the following parameters: field of view of 6‐cm circular pie; slice thickness of 0.1 mm; tube voltage of 90 kV; tube current of 9 mA; and scanning time of 17.5 seconds. The image was reformatted on the workstation (Volume Analyzer; SYNAPSE VINCENT, Fujifilm). The occlusion of the full cast crown attached to the right mandibular second molar was adjusted. There were no findings of marked inflammation in the surrounding gingiva. The probing depths of periodontal pockets around the right mandibular second molar were 3 mm.

## 3 TREATMENT

**FIGURE 2:**
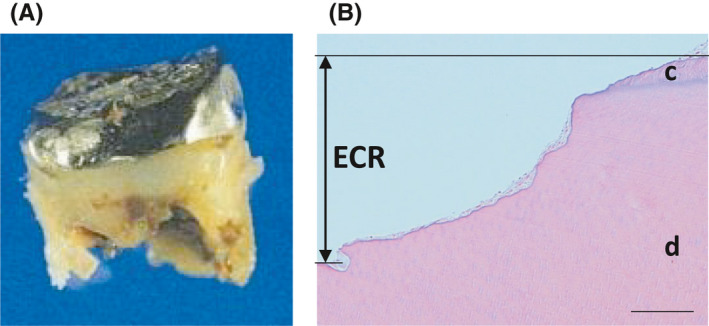
Macroscopic and histological views of the extracted right mandibular second molar. A, Macroscopic view. The root of the extracted tooth was largely resorbed. B, Histological view. The root was resorbed from the cementum side. Original magnifications: 10×; scale bar = 100 µm. Abbreviations: c, cementum; d, dentine; ECR, external cervical resorption

The right mandibular second and third molars were extracted. The right mandibular second molar was analyzed histologically by hematoxylin and eosin staining (Figure 2). The root had been resorbed from the cementum side, suggesting external root resorption (Figure 2B). The surrounding gingival tissue had inflammatory cell infiltration.

## 4 OUTCOME AND FOLLOW‐UP

**FIGURE 3:**
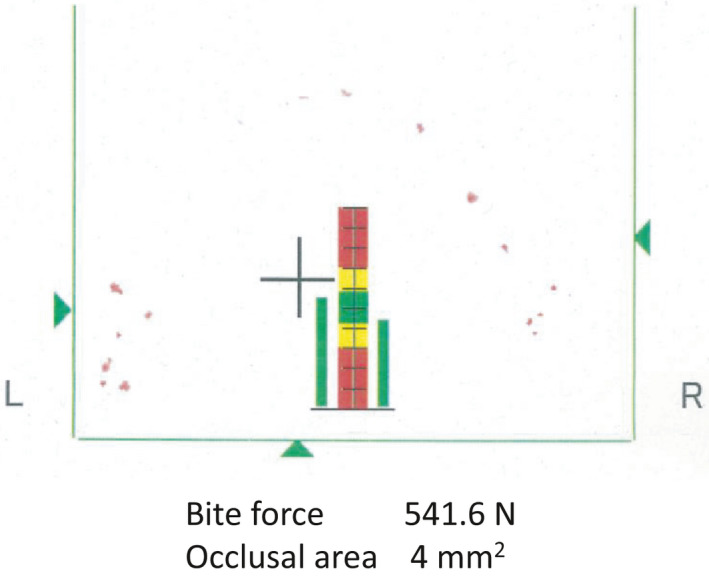
Results of the bite force test. Bite force and the occlusal area were 541.6 Newtons (N) and 4 mm^2^, respectively. Red dots show occlusal points

Healing after extraction was unremarkable and without complications. To investigate whether ECR was present in other teeth besides the right mandibular first molar, full‐mouth CBCT was performed and no other ECR was noted. A bite force test performed after extraction showed uniform force distribution, with no localized excess in bite force close to the right mandibular first molar (Figure 3). Since the patient was asymptomatic, he did not consent to treatment for the right mandibular first molar and requested to proceed with close observation instead. The right mandibular first molar will be monitored in subsequent visits to assess changes.

## 5 DISCUSSION

In the present case, ECR was observed in a patient with MOG antibody–associated disease. To our knowledge, there have been no reports of ECR in patients with MOG antibody–associated disease, and our patient is the first reported case in the literature. MOG antibody–associated disease may induce ECR via inflammation, orthodontic force, traumatic injury, internal bleaching, surgery, bruxism, or restoration. ^14^, ^15^, ^16^ Multiple root resorption has been reported in patients with autoimmune diseases, such as systemic scleroderma. ^11^, ^12^, ^13^ However, many aspects of the etiology of ECR and its association with systemic diseases remain unclear. ECR appears to be multifactorial in the majority of cases.^16^ The pathology of MOG antibody–associated disease may contribute to ECR. The levels of T‐helper 17 (Th17)–related cytokines, such as interleukin‐6 (IL‐6) and interleukin‐17 (IL‐17), are increased in the serum of MOG antibody–positive patients.^17^ This could be a potential mechanism of action as ECR is caused by odontoclastic cells, which are promoted by inflammatory cytokines; specifically, tumor necrosis factor alpha and IL‐6 are known to play a major mediatory role in odontoclastic cells.^18^ IL‐17 promotes the production of receptor activator of nuclear factor kappa beta, an osteoclast differentiation factor in mesenchymal supporting cells. Therefore, it is possible that the production of Th17‐related cytokines in MOG antibody–associated disease may be involved in ECR pathogenesis.

It has been reported that traumatic occlusion can cause progressive ECR.^19^ Since the full cast crown attached to the right mandibular second molar had already been adjusted, the right mandibular second molar may have previously received an excessive bite force. There is a possibility that the occlusion was involved in external root resorption at the right mandibular second molar. Occlusion may also be related to ECR of the right mandibular first molar. To investigate whether this could be relevant in the present case, the region bearing the bite force was assessed through a bite force test. Interestingly, there was no excessive bite force close to the right mandibular first molar, and therefore, the bite force was probably not responsible for ECR in the present case (Figure 3). In addition, as one of the causes of external root resorption of the right mandibular second molar, it is necessary to consider the pressure of the horizontally impacted right mandibular third molar. However, the pressure of this molar would not be involved in the ECR of the right mandibular first molar. In this case, it is likely that traumatic occlusion and pressure of the horizontally impacted right mandibular third molar did not play a role in the etiology of ECR of the right mandibular first molar, suggesting that the disordered production of inflammatory cytokines, such as IL‐17, may be the etiology of ECR in patients with MOG antibody–associated disease.

In recent studies, it was shown that other factors can be involved in the initiation of ECR. There are factors that can be clearly denied by patient anamnesis, such as a history of herpes zoster, autotransplantation, or playing wind instruments.^16^ On the other hand, other factors such as transmission of feline viruses and other viruses, which contribute to the initiation of ECR, can remain unclear by patient anamnesis.^20^ As mentioned above, ECR appears to be multifactorial in the majority of cases.^16^ Therefore, it is necessary to consider the possibility that ECR in this patient may be the result of not only the pathogenesis of MOG antibody–associated disease but also other factors. In addition, a new theory proposes that hypoxia plays an important role in ECR progression.^21^ Hypoxia is the driving force of angiogenesis, leading to the continuous development of highly vascularized granulation tissue accompanying ECR.^22^ Hypoxia within an ECR lesion can occur because of local alterations, which can be brought about by occlusal forces or inflammation.^23^ If inflammation due to the pathology of MOG antibody–associated disease is involved in hypoxia, it may have contributed to the occurrence of ECR in this case.

The use of CBCT proved to be useful in the diagnosis of ECR in our patient and could be essential for the proper diagnosis of ECR in patients with MOG antibody–associated disease. Dental radiography that is routinely performed is useful for the diagnosis of tooth and periodontal diseases. In fact, the obvious external root resorption of the right mandibular second molar was diagnosed using dental radiography. However, ECR of the right mandibular first molar was unclear on dental radiography and was instead incidentally discovered on CBCT, which was performed while designing the extraction plan for the right mandibular third molar. Given the possibility of ECR in other regions, close examination through full‐mouth CBCT was performed in our case, but no other ECR was noted. As the limitations of dental radiography are well documented and known to result in misdiagnosis and poor management of ECR, CBCT as additional testing could help diagnose patients with this disease.^24^

It is important to note that MOG antibody–associated disease is detectable in a minority of patients with inflammatory CNS demyelinating diseases such as multiple sclerosis (MS). MS is a chronic inflammatory disease of the CNS and is associated with demyelination and neurodegeneration. It has been suggested that patients with MS may be more likely to present with a range of oral and dental health problems and are at high risk of periodontal diseases.^25^, ^26^ An association between MS and temporomandibular joint arthrosis has also been reported.^27^ However, to our knowledge, ECR in a patient with MS has yet to be reported. Given the potential of ECR occurring in patients with MS, close dental and oral examination may also be needed in this patient group.

The treatment strategy for ECR differs depending on the region and the degree of resorption. CBCT is recommended to determine the best treatment for ECR.^28^ ECR can be treated with simple obturation of the lesions and endodontic treatment. However, if treatment of the lesions is not possible, dental extraction of the teeth is indicated.^28^ Patients with MOG antibody–associated disease may require treatment with a BP agent for the prevention of osteoporosis. Patients undergoing treatment with a BP agent are susceptible to MRONJ after invasive treatments, including dental extraction.^7^, ^8^ Therefore, oral cavity examination and CBCT are needed when considering treatment for patients with MOG antibody–associated disease before beginning treatment with BP agents and to help locate potentially treatable ECR lesions. In addition, long‐term follow‐up is required to observe whether ECR has progressed or appeared in other regions. In this case, the patient was informed of the need for treatment but did not agree to dental treatment. The duration of BP agent therapy continues to be a risk factor for developing MRONJ.^6^ Patients should be treated as soon as possible when the progression of the ECR lesion of the right mandibular first molar is observed during follow‐up. Also, it has been reported that BP agents are associated with ECR.^29^ It is necessary to consider the causes of adverse effects of BP agents when ECR occurs in patients with MOG antibody–associated disease.

The above case suggests that ECR may occur in patients with MOG antibody–associated disease, and CBCT is useful for the diagnosis of ECR in this case. ECR is often asymptomatic, and its detection by dental radiography is limited. Therefore, a CBCT examination is necessary, as shown in the case presented. Before considering the use of a BP agent for patients with MOG antibody–associated disease, full‐mouth CBCT is needed to examine for the presence or absence of ECR. The frequency of ECR in patients with MOG antibody–associated disease is unclear. Larger sample sizes are crucial to determine any potential association between ECR and MOG antibody–associated disease. MOG antibody–associated disease is a rare new neurological autoimmune disease. Many clinicians should pay attention to a possible association between ECR and MOG antibody–associated disease. Further clinical cases and basic studies are needed to clarify the relationship between MOG antibodies and ECR.

## CONFLICT OF INTEREST

None declared.

## AUTHOR CONTRIBUTIONS

SM, MU, YM, and MN: collected clinical data and produced the draft of the manuscript. KO, MK, and SM: made substantial contributions to conception and design. HM and HK: supervised the finalization of the manuscript. HF and MM: performed histological analyses. HN: performed surgical procedures. All authors: have read and approved the final manuscript.

## ETHICAL APPROVAL

A written informed consent was obtained from the patient for the publication of this report.

## Data Availability

The data that support the findings of this study are available on request from the corresponding author.
